# Influence of planting dates and fertilizer modules on yield of chrysanthemum and soil health

**DOI:** 10.1186/s12870-024-05241-y

**Published:** 2024-06-07

**Authors:** Sabhya Pathania, Sita Ram Dhiman, Bharati Kashyap, Anshul Kumar, Rajesh Kaushal, Rakesh Kumar Gupta, Ibrahim A. Saleh, Mohammad K. Okla, Mohamed Soliman Elshikh

**Affiliations:** 1 Department of Floriculture and Landscape Architecture, Dr YS Parmar University of Horticulture and Forestry, Nauni, Solan, 173230 India; 2 Department of Floriculture and Landscape Architecture, Dr YS Parmar University of Horticulture and Forestry, Nauni, Solan, 173230 India; 3 Department of Soil Science and Water Management, Dr YS Parmar University of Horticulture and Forestry, Nauni, Solan, 173230 India; 4 Department of Basic Sciences, Dr YS Parmar University of Horticulture and Forestry, Nauni, Solan, 173230 India; 5 Faculty of Science, Zarqa University, Zarqa, 13110 Jordan; 6 Botany and Microbiology Department, College of Science, King Saud University, P.O. Box 2455, Riyadh, 11451 Saudi Arabia

**Keywords:** Chrysanthemum, Inorganic fertilization, Jeevamrit, Microbial population, Planting dates, Yield and quality

## Abstract

**Background:**

Optimum planting date and appropriate fertilizer module are essential facets of chrysanthemum cultivation, to enhance quality yield, and improve soil health. A field-based study was undertaken over multiple growing seasons in 2022 and 2023, where six different planting dates, viz., P_1_:June 15, P_2_:June 30, P_3_:July 15, P_4_:July 30, P_5_:August 15 and P_6_:August 30 and two fertilizer modules, FM_1_:Jeevamrit @ 30 ml plant^−1^ and FM_2_:NPK @ 30 g m^−2^ were systematically examined using a Randomized Block Design (factorial), replicated thrice.

**Results:**

P_6_ planting resulted in early bud formation (44.03 days) and harvesting stage (90.78 days). Maximum plant height (79.44 cm), plant spread (34.04 cm), cut stem length (68.40 cm), flower diameter (7.83 cm), stem strength (19.38˚), vase life (14.90 days), flowering duration (24.08 days), available soil N (314 kg ha^−1^), available P (37 kg ha^−1^), available K (347 kg ha^−1^), bacterial count (124.87 × 10^7^ cfu g^−1^ soil), actinomycetes count (60.72 × 10^2^ cfu g^−1^ soil), fungal count (30.95 × 10^2^ cfu g^−1^ soil), microbial biomass (48.79 µg g^−1^ soil), dehydrogenase enzyme (3.64 mg TPF h^−1^ g^−1^ soil) and phosphatase enzyme (23.79 mol PNP h^−1^ g^−1^ soil) was recorded in P_1_ planting. Among the fertilization module, minimum days to bud formation (74.94 days) and days to reach the harvesting stage (120.95 days) were recorded with the application of NPK @30 g m^−2^. However, maximum plant height (60.62 cm), plant spread (23.10 cm), number of cut stems m^−2^ (43.88), cut stem length (51.34 cm), flower diameter (6.92 cm), stem strength (21.24˚), flowering duration (21.75 days), available soil N (317 kg ha^−1^), available P (37 kg ha^−1^) and available K (349 kg ha^−1^) were also recorded with the application of NPK @300 kg ha^−1^. Maximum vase life (13.87 days), OC (1.13%), bacterial count (131.65 × 10^7^ cfu g^−1^ soil), actinomycetes count (60.89 × 10^2^ cfu g^−1^ soil), fungal count (31.11 × 10^2^ cfu g^−1^ soil), microbial biomass (51.27 µg g^−1^ soil), dehydrogenase enzyme (3.77 mg TPF h^−1^ g^−1^ soil) and phosphatase enzyme (21.72 mol PNP h^−1^ g^−1^ soil) were observed with the application of Jeevamrit @ 30 ml plant^−1^.

**Conclusion:**

Early planting (P_1_) and inorganic fertilization (NPK @ 30 g m^−2^) resulted in improved yield and soil macronutrient content. The soil microbial population and enzymatic activity were improved with the jeevamrit application. This approach highlights the potential for improved yield and soil health in chrysanthemum cultivation, promoting a more eco-friendly and economically viable agricultural model.

## Introduction

*Chrysanthemum* (*Dendranthema grandiflora* Tzvelev) is a highly prized ornamental plant globally, valued for its extensive variability in cultivars showcasing various colours, sizes, and flower patterns [1]. Originating in Asia and North-eastern Europe, *Chrysanthemum* has been under cultivation for over 1600 years [2]. It is widely grown for ornamental, culinary, and medicinal purposes worldwide. India's diverse agro-climatic conditions provide an ideal environment for year-round *Chrysanthemum* cultivation, spanning across various regions [3]. Due to its extensive farming sector, India has a competitive advantage over other industrialised countries in meeting the significant global demand for produce in large numbers at a significantly lower cost. Current trends in the *Chrysanthemum* industry emphasise the enhancement of flower quality and the development of environmentally sustainable production systems. To attain these objectives and expedite *Chrysanthemum* production, continued innovation is essential in refining fertilization strategies and advancing other cultivation techniques.

Growers have to produce the right amount and quality at the right moment under the pressure of the market throughout the year [4]. *Chrysanthemum*, characterised as a short-day plant, exhibits a limited availability period of approximately three months [5]. Planting them simultaneously leads to market oversupply, consequently causing a depreciation in market prices [6]. Scheduling of planting dates helps in regulating the flowering duration to mitigate such market fluctuations and fetch better market prices along with an increase in demand [7]. So different planting dates were evaluated for quality cut stems production of *Chrysanthemum*.

Along with planting dates, the optimum application of fertilizers is an important element in *Chrysanthemum* cultivation for plant growth, and soil function sustainability. *Chrysanthemum*, being a high-demanding crop, necessitates significant quantities of crucial nutrients, with nitrogen, phosphorous, and potassium being particularly essential. In conventional agricultural practices, these nutrient requirements are typically fulfilled through the application of chemical fertilizers [8]. However, the widespread use of these chemical fertilizers has led to a decline in soil fertility in modern agriculture, contributing to soil acidification in India and posing a threat to the long-term sustainability of Indian agriculture, thereby affecting the livelihoods of farming communities [9]. It is highly advised to apply organic manures to the soil in order to reduce the negative impacts of inorganic fertilizers. In addition to traditional organic formulations, fermented liquid bio-formulations like Jeevamrit have gained acceptance for enhancing soil fertility and productivity [10]. Jeevamrit is a mixture of urine, gram flour, jaggery, and dung that is rich in nitrogen and carbon. It is used to stimulate plant development, enhance root biomass, and support soil microbes. The high microbial count in Jeevamrit, possibly attributed to its constituents, acts as a stimulant for soil microbial activity, contributing to a healthier soil ecosystem. Several workers had studied the effects of chemical fertilizers, organic fertilizers and their integration in *Chrysanthemum* [11–14] but the sidewise comparison of chemical fertilizer and biostimulants, especially Jeevamrit is lacking. The present study was aimed to evaluate the effect of different fertilizer regimes at different planting dates on yield, quality, as well as soil health. In addition, analyzing variations in soil enzyme activities (including phosphatase and dehydrogenase), microbial biomass, fungal, bacterial, and actinomycetes populations in the rhizosphere can enhance our understanding of microbial activity changes in *Chrysanthemum* rhizosphere under different treatments of planting dates and fertilization modules.

The objective of this study is to characterize and examine the growth and yield dynamics of *Chrysanthemum* in response to planting date (season) and fertilization regime, as well as their combined effects. Six experiments were conducted, encompassing planting dates across different seasons, each paired with two distinct fertilizer regimes.

## Materials and methods

### Experimental site and plant material

**Fig. 1:**
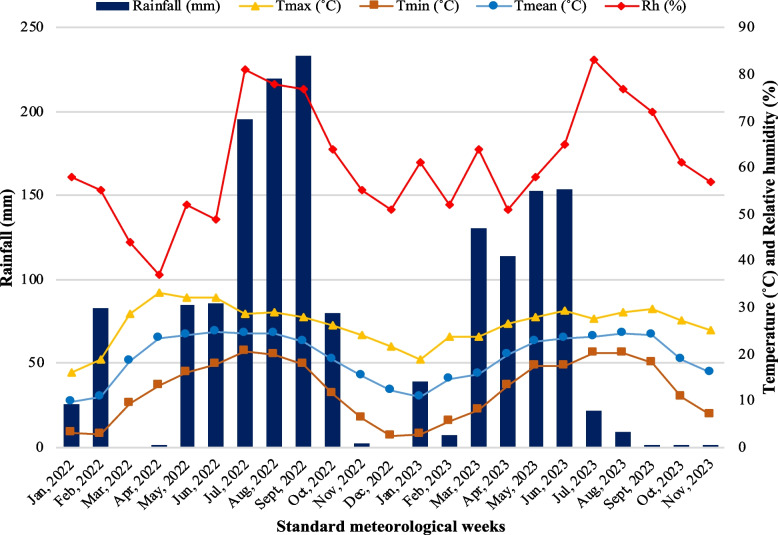
Agrometeorological data during the experimental period (2022 and 2023)

The experimental site was, the Dr. Y.S. Parmar University of Horticulture and Forestry Nauni, Solan (Himachal Pradesh) experimental farm, which is situated at 30°520 latitude N and 77°110 longitude E at an elevation of 1260 m amsl in the mid-hills zone, served as the site of the field experiment for two consecutive growing seasons in 2022 and 2023. About 75% of the region's average rainfall (1115 mm), occurs during the monsoon, which runs from mid-June to mid-September. Fig. 1 displays the meteorological data collected during the experiment.

At the experimental farm, four-week-old rooted cuttings of the cultivar ‘Solan Shringar’ were prepared. On a six-inch raised bed measuring 1 m^−2^, uniformly sized rooted cuttings were space planted at 30 × 30 cm, supporting 9 plants m^−2^. In order to prepare the bed, the soil was dug down to a depth of 30 cm, and well-rotted FYM (farm yard manure) @ 5 kg m^−2^ was incorporated into the soil. Before the experiment began, the soil's chemical characteristics were assessed (Table 1). The experimental field had sandy loam soil with good drainage and optimum water retention capacity.

**Table 1:** Initial soil characteristics

Particular	Method employed	Soil Status	Reference(s)
pH	1:2 (soil: water) suspension, with the help of digital pH meter	6.85	[15]
EC	1:2 (soil: water) suspension, with the help of digital EC meter	0.34	[15]
Organic Carbon	Rapid titration method	0.92 (%)	[16]
Available N	Alkaline potassium permanganate method	292.76 (kg ha^−1^)	[17]
Available P	Olsen’s method	27.48 (kg ha^−1^)	[18]
Available K	Ammonium acetate method	315.84 (kg ha^−1^)	[19]

### Design of experiment and chemical analysis of Jeevamrit

Twelve treatments were used in the experiment (Table 2), which was set up in a randomized block design (RBD) factorial, with three replications. The experiment consists of a factorial design, viz., planting dates and fertilization module. Across the period from June 15 to August 30, 2022–2023, planting was executed on six different dates, viz., P_1_: June 15, P_2_: June 30, P_3_: July 15, P_4_: July 30, P_5_: August 15, and P_6_: August 30, maintaining a 15-day interval time period. June & July planting were done accommodating 9 plants m^−2^ at a spacing of 30 × 30 cm. However, August planting was done accommodating 49 plants m^−2^ and grown without pinching. The variation in the number of plants among different planting dates was to balance the total biomass, since later plantings do not respond to pinching due to the commencement of short days. For the inorganic fertilization module, after the preparation of beds, the basal doze of chemical fertilizer was incorporated in the beds through 300 kg ha^−1^ each of NPK. Half of the nitrogen (N) dosage, the complete phosphorus (P) dosage, and the entire potassium (K) dosage were applied at the initiation of bed preparation as basal dose. The remaining half of the nitrogen (150 kg ha^−1^) was incorporated into the soil 45 days after planting. Urea, murate of potash (MOP) and single super phosphate (SSP) were used as different nutrient sources. For the organic fertilization module, drenching with Jeevamrit was done at a rate of 30 ml plant^−1^. Jeevamrit was prepared at the experimental farm by mixing 10 L of cow urine, 10 kg of cow dung, 2 kg of jaggery, 2 kg of pulse flour, 0.5 kg of soil from beneath a tree, and 200 L of water in a plastic drum covered with a damp jute bag, which was kept in a shaded area. The mixture was stirred twice daily in a clockwise direction. On the fifth day, the solution was filtered, and the filtrate was used for soil application. The application was started at 30 days after transplanting, and drenching was done at 30 ml per plant at 1:4 dilutions. The nutrient status, microbial load and enzymatic activity of the Jeevamrit are given in Table 3. Standard cultural operations were followed to raise a healthy crop throughout the experiment. Pinching was done to induce lateral branches at 5–6 leaf stage. Irrigation was done twice a week in winters and on an alternate day during summers (upto 8 cm depth). Plants were uprooted after completion of the experiment in each year and fresh planting was done for the second year experiment.

**Table 2:** Treatment details for experimental field

Treatment	Treatment code	Detail
**Planting dates**	P_1_	June 15
	P_2_	June 30
	P_3_	July 15
	P_4_	July 30
	P_5_	August 15
	P_6_	August 30
**Fertilizer modules**	FM_1_	Jeevamrit @ 30 ml/plant
	FM_2_	NPK @ 30 g/m^2^

**Table 3:** Nutrient status and microbial load in Jeevamrit

Parameter	Nutrient Status
pH	8.2
EC (dsm^−1^)	5.7
Total nitrogen (%)	4.3
Total phosphorous (mg kg^−1^)	168.4
Total potassium (mg kg^−1^)	259.6
Bacteria (cfu)	16.8 × 10^5^
Fungi (cfu)	11.5 × 10^3^
Actinomycetes (cfu)	7.2 × 10^3^
Total zinc (mg kg^−1^)	2.96
Total copper (mg kg^−1^)	0.69
Total iron (mg kg^−1^)	16.84
Total manganese (mg kg^−1^)	3.52
Phosphatase (µg mL^−1^)	7.13
Dehydrogenase (µg mL^−1^)	2.87

### Vegetative and flowering attributes

For every replication and treatment, five plants were randomly selected and all the vegetative parameters (plant height and spread) and flowering parameters (days for bud formation, for harvesting stage, number of cut stems per plant, number of cut stems per m^2^, cut flower stem length, flower diameter, stem strength, vase life, and duration of flowering) were noted from each date of planting (P_1_-P_6_) at proper stage of data collection. Plant spread was recorded at the time of harvesting of first cut stems as average distance between the outer most side shoot in east to west direction and the distance between outermost side shoot in north to south direction [29]. Stem strength was noted by measuring the level of sturdiness and was taken by holding the cut stem of 30 cm horizontally above the cut end and measuring the angle of deviation of the flower head below the horizontal plane with the natural curvature of the stem. Vase life was recorded from the date of placing flowers in the vase containing distilled water to the stage untill they remained presentable [29]. Every tagged plant's cut stem count, including the number of stems per plant and per m^2^, was recorded and the average result was calculated. A digital vernier calliper was used for parameters such as flower diameter.

### Soil chemical properties

Soil samples, composite in nature, were collected from depths ranging from 0 to 15 cm before the experiment’s initiation. Post-study completion, samples of soil were collected to analyze the various soil parameters from each treatment. In accordance with [15], soil pH and electrical conductivity (EC) were measured. Organic carbon content was determined following the method described by [16], available nitrogen was assessed using the [17] method, available phosphorus was determined using the [18] procedure, and available potassium was measured using the method outlined by [19]. These measurements were made consecutively over a two-year period.

### Microbiological properties of soil

The data on microbiological properties of soil were analysed after completion of trial from each treatement. The quantification of viable microbes was conducted on nutrient agar (NA) for bacterial count, potato dextrose agar medium (PDA) for fungal count, and Kenknight and Munaier's medium (KNM) for actinomycetes count, using the serial dilution standard spread plate technique [20]. The population was expressed in terms of colony forming units (cfu) per gram of soil and the assessment of microbial biomass-C was done using the soil fumigation extraction method [21]:

$$MB\;-\;C\;\left(\mathrm\mu g\;g^{-1}\;\mathrm{soil}\right)\;=\;\frac{EC\;\left(F\right)\;-\;EC\;\left(UF\right)}K$$

Where, K = 0.25 ± 0.05 (factor which represents the efficiency of extraction of microbial biomass carbon)

EC (F) = Total amount of extractable carbon in fumigated soil samples.

EC (UF) = Total amount of extractable carbon in un-fumigated soil samples.

The 2,3,5-triphenyl tetrazolium chloride (TTC) reduction method was used to estimate the dehydrogenase activity in liquid formulations [22]. The estimation of phosphatase activity was carried out using the procedure outlined by [23].

### Statistical analysis

The data obtained from different treatments during the research was analysed using SPSS version 16.0 software (SPSS Inc., Chicago, IL, USA). Employing a randomized block design (RBD), a one-way analysis of variance (ANOVA) was performed on the combined data, and the treatments were compared at the 0.05% significance level.

## Results

### Vegetative parameters

Data on plant height annd plant spread varied significantly by planting dates and fertilization modules, interaction effect of plant height (pooled analysis) and plant spread (2021–22, 2022–23 and pooled analysis was significant. P_1_ planting resulted in maximum plant height (74.73 cm) and plant spread (34.04 cm), whereas, minimum was recorded in P_6_ planting (34.69 cm and 6.62 cm, respectively). Optimum nutrition is essential for plant growth. Fertilization modules have significant effect on plant growth. FM_2_ module resulted in maximum plant height and plant spread (60.62 cm and 23.10 cm, respectively). P_1_ planting and FM_2_ module resulted in improved vegetative characters i.e. plant height and plant spread.

### Flowering attributes

Flowering parameters were significantly influenced by date of planting and fertilization modules (Table 4). Data on number of days taken for bud formation, days taken to reach the harvesting stage and duration of flowering varied significantly by planting dates and fertilization modules, however their interaction was effect was found non significant. The minimum number of days required to bud formation (44.03 days) and to reach the harvesting stage (90.78 days) was recorded in P_6_ planting while the maximum was recorded in P_1_ planting (109.15 and 158.68 days, respectively). An extended duration of flowering (24.08 days) was observed in P_1_ planting and P_6_ planting resulted in the shortest duration of flowering (24.08 days). Plants grown under FM_2_ module required the shortest duration to bud formation (74.94 days) and to reach the harvesting stage (123.79), whereas, maximum days required to bud formation (77.02 days) and to reach the harvesting stage (125.72 days) were recorded in FM_1_. The maximum duration of flowering (21.75 days) was recorded in FM_2_ and the minimum duration of flowering (20.06 days) was recorded when the plants were grown under FM_1_. P_6_ planting and FM_2_ fertilization advanced the bud formation and harvesting stage. Extended flowering duration was observed in P_1_ and FM_2_.

**Table 4:** Effect of planting dates and fertilization on vegetative parameters

Treatments	Plant height (cm)			Plant Spread (cm)		
	2021–22	2022–23	Pooled Mean	2021–22	2022–23	Pooled Mean
**Planting Dates**						
** P**_**1**_	74.73^a^	84.15^a^	79.44^a^	31.79^a^	36.28^a^	34.04^a^
** P**_**2**_	69.32^b^	75.62^b^	72.47^b^	29.49^b^	34.02^b^	31.75^b^
** P**_**3**_	61.43^c^	67.51^c^	64.47^c^	26.70^c^	30.23^c^	28.47^c^
** P**_**4**_	50.65^d^	55.83^d^	53.24^d^	22.84^d^	26.63^d^	24.74^d^
** P**_**5**_	40.09^e^	45.52^e^	42.81^e^	6.92^e^	7.76^e^	7.34^e^
** P**_**6**_	30.37^f^	39.00^f^	34.69^f^	6.21^e^	7.02^e^	6.62^e^
** Significance**	*	*	*	*	*	*
** SE**	0.49	0.14	0.24	0.25	0.256	0.25
**Fertilizer modules**						
** FM**_**1**_	52.00^b^	58.18^b^	55.09^b^	19.71^b^	22.72^b^	21.21^b^
** FM**_**2**_	56.86^a^	64.37^a^	60.62^a^	21.61^a^	24.60^a^	23.10^a^
** Significance**	*	*	*	*	*	*
** SE**	0.85	0.25	0.42	0.44	0.444	0.432
**Interactions**						
** P1xFM1**	73.28	81.65	77.47^b^	30.05^b^	34.53^b^	32.29^b^
** P2xFM1**	67.73	72.61	70.17^d^	28.01^c^	32.44^c^	30.22^c^
** P3xFM1**	58.31	64.38	61.34^f^	25.43^d^	29.07^d^	27.25^d^
** P4xFM1**	46.66	52.36	49.51^ h^	21.59^e^	25.59^e^	23.59^e^
** P5xFM1**	36.71	42.02	39.36^j^	6.91^f^	7.70^f^	7.30^f^
** P6xFM1**	29.32	36.04	32.68^ l^	6.25^f^	7.00^f^	6.60^f^
** P1xFM2**	76.18	86.64	81.41^a^	33.52^a^	38.04^a^	35.78^a^
** P2xFM2**	70.913	78.63	74.77^c^	30.97^b^	35.60^b^	33.28^b^
** P3xFM2**	64.553	70.65	67.60^e^	27.97^c^	31.39^c^	29.68^c^
** P4xFM2**	54.647	59.31	56.98^ g^	24.09^d^	27.67^d^	25.88^d^
** P5xFM2**	43.467	49.03	46.25^i^	6.94^f^	7.83^f^	7.38^f^
** P6xFM2**	31.42	41.96	36.69^ k^	6.16^f^	7.05^f^	6.63^f^
** Significance**	NS	NS	*	*	*	*
** SE**	1.20	0.35	0.59	0.62	0.628	0.611

The values in each column that are preceded by the same letter are not significantly different from one another (DMRT, *p* ≤ 0.05)

Where *SE* is standard error, *NS* is Non Significant and * is significant at 5% significance

### Yield and quality attributes

Data on number of cut stems per plant, number of cut stems per m^2^, flower diameter (cm), cut flower stem length,stem strength and vase life varied significantly by planting dates, however, in case of fertilization module flower diameter, cut flower stem length and vase life varied significantly and number of cut stems per plant, number of cut stems per m^2^, stem strength had non-significant effect. Interaction effect was found to be non-significant for all the yield and quality attributes except flower diameter. The observation of P_1_ planting revealed the highest count of cut stems per individual plant (5.22), whereas P_5_ and P_6_ observed the minimum number of cut stems (1.00). The maximum number of cut stems per m^2^ (49.00) was recorded in P_5_ and P_6_ plantings, on the contrary, the minimum was found in P_4_ planting (35.10). Maximum cut flower stem length (68.40 cm), flower diameter (7.83 cm), stem strength (19.38˚) and vase life (14.90 days) were recorded in P_1_ planting. P_6_ planting resulted in the minimum number of cut stems per plant (5.22), number of cut stems per m^2^ (46.95), cut flower stem length (68.40 cm), flower diameter (7.83 cm), stem strength (19.38˚) and vase life (14.90 days).

The maximum number of cut stems per plant (3.39), number of cut stems per m^2^ (43.88), cut flower stem length (51.34 cm), flower diameter (6.92 cm) and stem strength (21.24) were recorded in FM_2_. The minimum number of cut stems per plant (3.31), number of cut stems per m^2^ (43.13), cut flower stem length (45.80 cm), flower diameter (6.33 cm) and stem strength (21.95˚) was recorded when the plants were grown under FM_1_. Maximum vase life (13.87 days) was observed in FM_1_, whereas, minimum was in FM_2_ (12.95 days). P_5_ and P_6_ plantings along with FM_2_ resulted in a higher number of cut stems per m^2^. P_1_ planting and FM_2_ improved yield and quality attributes like the number of cut stems per plant, cut flower stem length, flower diameter, stem strength and vase life.

### Macro-nutrient content in the soil

The recorded data illustrating the impact of both planting dates and modules of fertilization on soil macronutrient content is presented in Table 5. Data on available nitrogen, available phosphorous and available potassium varied significantly for planting dates and fertilization modules, however their interaction effect was found non significant. Available nitrogen (314.36 kg ha^−1^), phosphorous (36.61 kg ha^−1^) and potassium content (346.56 kg ha^−1^) was highest in soil under P_1_ planting and lowest was recorded in P_6_ planting (304.86, 33.10 and 337.36 kg ha^−1^, respectively). Plants grown under FM_2_ recorded maximum available nitrogen (316.84 kg ha^−1^), phosphorous (36.86 kg ha^−1^) and potassium (348.91 kg ha^−1^) in soil, in contrast, a minimum was observed in FM_1_ (302.62, 32.55 and 334.07 kg ha^−1^, respectively). P_1_ planting and FM_2_ resulted in maximum macro-nutrient content in the soil.

**Table 5.** Effect of planting dates and fertilization on flowering attributes

Treatments	Number of days taken for bud formation (days)			Days taken to reach the harvesting stage (days)			Duration of Flowering (days)		
	2021–22	2022–23	Pooled Mean	2021–22	2022–23	Pooled Mean	2021–22	2022–23	Pooled Mean
**Planting Dates**									
** P1**	112.20^a^	106.10^a^	109.15^a^	161.17^a^	156.20^a^	158.68^a^	25.50^a^	22.67^a^	24.08^a^
** P2**	98.60^b^	92.87^b^	95.73^b^	148.20^b^	142.17^b^	145.18^b^	24.33^ab^	21.50^ab^	22.92^ab^
** P3**	83.33^c^	77.37^c^	80.35^c^	134.47^c^	128.63^c^	131.55^c^	23.33^abc^	20.33^abc^	21.83^abc^
** P4**	72.07^d^	66.13^d^	69.10^d^	120.25^d^	116.40^d^	118.33^d^	21.67^bc^	18.17^bcd^	19.92^cd^
** P5**	60.50^e^	54.57^e^	57.53^e^	106.68^e^	101.33^e^	104.01^e^	20.50^cd^	17.50^bcde^	19.00^e^
** P6**	46.97^f^	41.10^f^	44.03^f^	93.18^f^	88.37^f^	90.78^f^	19.50^d^	15.83^e^	17.67^e^
** Significance**	*	*	*	*	*	*	*	*	*
** SE**	0.06	0.11	0.07	0.18	0.15	0.13	0.51	0.69	0.55
**Fertilizer modules**									
** FM1**	80.02^a^	74.02^a^	77.02^a^	128.03^a^	123.42^a^	125.72^a^	21.67^b^	18.44^b^	20.06^b^
** FM2**	77.87^b^	72.02^b^	74.94^b^	126.62^b^	120.95^b^	123.79^b^	23.28^a^	20.22^a^	21.75^a^
** Significance**	*	*	*	*	*	*	*	NS	*
** SE**	0.11	0.19	0.12	0.31	0.26	0.23	0.88	1.20	0.95
**Interactions**									
** P1xFM1**	113.40	107.27	110.33	162.27	157.33	159.80	24.67	22.33	23.50
** P2xFM1**	99.60	93.80	96.70	149.13	142.33	145.73	23.67	20.33	22.00
** P3xFM1**	84.47	78.73	81.60	135.13	129.00	132.07	22.33	19.67	21.00
** P4xFM1**	73.13	67.00	70.07	120.43	118.77	119.60	20.67	17.00	18.83
** P5xFM1**	61.67	55.67	58.67	107.07	103.40	105.23	19.67	16.67	18.17
** P6xFM1**	47.87	41.67	44.77	94.13	89.67	91.90	19.00	14.67	16.83
** P1xFM2**	111.00	104.93	107.97	160.07	155.07	157.57	26.33	23.00	24.67
** P2xFM2**	97.60	91.93	94.77	147.27	142.00	144.63	25.00	22.67	23.83
** P3xFM2**	82.20	76.00	79.10	133.80	128.27	131.03	24.33	21.00	22.67
** P4xFM2**	71.00	65.27	68.13	120.07	114.03	117.05	22.67	19.33	21.00
** P5xFM2**	59.33	53.47	56.40	106.30	99.27	102.78	21.33	18.33	19.83
** P6xFM2**	46.07	40.53	43.30	92.23	87.07	89.65	20.00	17.00	18.50
** Significance**	NS	NS	NS	NS	NS	NS	NS	NS	NS
** SE**	0.16	0.28	0.17	0.44	0.37	0.32	1.24	1.69	1.35

The values in each column that are preceded by the same letter are not significantly different from one another (DMRT, *p*≤0.05)

Where *SE *is standard error, *NS *is Non Significant and * is significant at 5% significance

### Soil chemical properties

During both of the study years, fertilizer and date planting had non-significant effects on the soil pH and soil EC values (Table 6). OC varied significantly for planting dates and fertilization modules. However interaction effects was found non significant. The pH ranged from 6.87–6.95, whereas the average EC value varied from 0.37—0.41 ds m^−1^. Maximum organic carbon in soil (1.13%) was recorded in P_1_, in contrast, minimum (1.05%) in P_6_. FM_1_ observed maximum organic carbon in soil (1.14%) over FM_2_ (1.07%).

**Table 6.** Effect of planting dates and fertilization on yield and quality attributes

Treatments	Number of cut stems per plant			Number of cut stems per m^2^			Flower diameter (cm)			Cut Flower Stem Length (cm)			Stem Strength ( ˚)			Vase Life (days)		
	2021–22	2022–23	Pooled Mean	2021–22	2022–23	Pooled Mean	2021–22	2022–23	Pooled Mean	2021–22	2022–23	Pooled Mean	2021–22	2022–23	Pooled Mean	2021–22	2022–23	Pooled Mean
**Planting Dates**																		
** P1**	5.00^a^	5.43^a^	5.22^q^	45.00^a^	48.90^a^	46.95^a^	7.60^a^	8.05^a^	7.83^a^	63.99^a^	72.8^a^	68.4^a^	19.57^b^	19.20^b^	19.38^b^	14.47^a^	15.33^a^	14.90^a^
** P2**	4.53^b^	4.87^b^	4.70^b^	40.80^b^	43.80^b^	42.30^b^	6.94^b^	7.57^ab^	7.26^b^	58.65^b^	65.04^b^	61.85^b^	20.17^b^	19.70^b^	19.93^b^	13.93^b^	14.57^b^	14.25^b^
** P3**	4.10^c^	4.50^c^	4.30^c^	36.90^c^	40.50^c^	38.70^c^	6.57^c^	7.16^bc^	6.87^c^	51.11^c^	55.6^c^	53.36^c^	20.37^b^	19.93^b^	20.15^b^	13.30^c^	14.00^c^	13.65^c^
** P4**	3.83^d^	3.97^d^	3.90^d^	34.50^d^	35.70^d^	35.10^d^	6.11^d^	6.72^cd^	6.41^d^	39.72^d^	45.87^d^	42.8^d^	21.97^b^	20.83^b^	21.40^b^	12.6^d^	13.47^d^	13.03^d^
** P5**	1.00^e^	1.00^e^	1.00^e^	49.00^e^	49.00^e^	49.00^e^	5.69^e^	6.33^de^	6.01^e^	32.58^e^	37.46^e^	35.02^e^	24.23^a^	23.83^a^	24.03^a^	12.13^d^	13.00^e^	12.57^e^
** P6**	1.00^e^	1.00^e^	1.00^e^	49.00^e^	49.00^e^	49.00^e^	5.03^f^	5.72^e^	5.37^f^	26.99^f^	33.02^f^	30.00^f^	24.93^a^	24.40^a^	24.67^a^	11.57^e^	12.57^f^	12.07^f^
** Significance**	*	*	*	*	*	*	*	*	*	*	*	*	*	*	*	*	*	*
** SE**	0.05	0.05	0.03	0.42	0.41	0.30	0.08	0.04	0.04	0.32	0.17	0.21	0.31	0.30	0.29	0.07	0.04	0.04
**Fertilizer modules**																		
** FM1**	3.20	3.42	3.31	42.13	44.13	43.13	6.10^b^	6.57^b^	6.33^b^	42.50^b^	49.10^b^	45.80^b^	22.20	21.70	21.95	13.44^a^	14.30^a^	13.87^a^
** FM2**	3.29	3.50	3.39	42.93	44.83	43.88	6.55^a^	7.28^a^	6.92^a^	48.51^a^	54.17^a^	51.34^a^	21.54	20.93	21.24	12.56^b^	13.34^b^	12.95^b^
** Significance**	NS	NS	NS	NS	NS	NS	*	*	*	*	*	*	NS	NS	NS	0.2	*	*
** SE**	0.08	0.08	0.06	0.73	0.71	0.53	0.13	0.07	0.07	0.55	0.29	0.36	0.53	0.51	0.50	0.12	0.06	0.07
**Interactions**																		
** P1xFM1**	4.87	5.33	5.10	43.80	48.00	45.90	7.12^bc^	7.47^c^	7.3^c^	62.29	70.57	66.43	19.93	19.73	19.83	14.80	15.87	15.33
** P2xFM1**	4.47	4.80	4.63	40.20	43.20	41.70	6.54^cd^	7.05^d^	6.79^d^	55.91	62.42	59.16	20.33	19.73	20.03	14.47	15.00	14.73
** P3xFM1**	4.07	4.53	4.30	36.60	40.80	38.70	6.12^def^	6.66^e^	6.39^e^	46.74	53.18	49.96	20.60	20.07	20.33	13.67	14.47	14.07
** P4xFM1**	3.80	3.87	3.83	34.20	34.80	34.50	5.84^efg^	6.44^ef^	6.14^ef^	35.75	43.75	39.75	21.80	21.00	21.40	13.07	13.93	13.50
** P5xFM1**	1.00	1.00	1.00	49.00	49.00	49.00	5.57^fg^	6.28^f^	5.93^f^	29.52	34.27	31.90	25.07	24.67	24.87	12.60	13.40	13.00
** P6xFM1**	1.00	1.00	1.00	49.00	49.00	49.00	4.67^h^	5.49^h^	5.44^g^	24.77	30.38	27.57	25.47	25.00	25.23	12.07	13.13	12.60
** P1xFM2**	5.13	5.53	5.33	46.20	49.80	48.00	8.09^a^	8.63^a^	8.36^a^	65.68	75.03	70.36	19.20	18.67	18.93	14.13	14.80	14.47
** P2xFM2**	4.60	4.93	4.77	41.40	44.40	42.90	7.35^b^	8.09^b^	7.72^b^	61.39	67.66	64.53	20.00	19.67	19.83	13.40	14.13	13.77
** P3xFM2**	4.13	4.47	4.30	37.20	40.20	38.70	7.02^bc^	7.66^c^	7.34^c^	55.48	58.02	56.75	20.13	19.80	19.97	12.93	13.53	13.23
** P4xFM2**	3.87	4.07	3.97	34.80	36.60	35.70	6.37^de^	6.99^d^	6.68^d^	43.69	47.99	45.85	22.13	20.67	21.40	12.13	13.00	12.57
** P5xFM2**	1.00	1.00	1.00	49.00	49.00	49.00	5.8^efg^	6.38^ef^	6.09^ef^	35.63	40.65	38.14	23.40	23.00	23.20	11.67	12.60	12.13
** P6xFM2**	1.00	1.00	1.00	49.00	49.00	49.00	5.39^g^	5.95^g^	5.31^g^	29.21	35.65	32.43	24.40	23.80	24.10	11.07	12.00	11.53
** Significance**	NS	NS	NS	NS	NS	NS	*	*	*	NS	NS	NS	NS	NS	NS	NS	NS	NS
** SE**	0.12	0.11	0.08	1.03	1.00	0.74	0.19	0.10	0.10	0.78	0.41	0.51	0.76	0.72	0.70	0.16	0.09	0.10

The values in each column that are preceded by the same letter are not significantly different from one another (DMRT, *p* ≤ 0.05)

Where *SE *is standard error, *NS* is Non Significant and * is significant at 5% significance

### Soil microbial properties

#### Viable microbial Count

Bacterial count in soil varied significantly for planting dates and fertilization modules. Fungal count and actinomycetes count in soil varied significantly for fertilization module however, planting dates had non-significant effect. The interaction effect for the soil microbial properties was also found non-significant. The average viable bacterial count reached its peak (124.87 × 10^7^ cfu g^−1^ soil) in P_1_, the lowest viable bacterial count (114.90 × 10^7^ cfu g^−1^ soil) was recorded in P_6_ and P_4_. Notably, the planting dates did not significantly influence the viable actinomycetes and fungal count in the soil. P_1_ planting recorded the maximum microbial biomass in soil, on the contrary P_6_ recorded the minimum (Table 7).

**Table 7:** Effect of planting dates and fertilization on macro-nutrient content in the soil

	Available Nitrogen (kg ha^−1^)			Available Phosphorous (kg ha^−1^)			Available Potassium (kg ha^−1^)		
Treatments	2021–22	2022–23	Pooled Mean	2021–22	2022–23	Pooled Mean	2021–22	2022–23	Pooled Mean
Planting Dates									
P1	311.86^a^	316.85^a^	314.36^a^	35.77^a^	37.44^a^	36.61^a^	346.48^a^	346.64^a^	346.56^a^
P2	310.84^ab^	315.04^ab^	312.95^ab^	34.91^a^	36.41^ab^	35.66^ab^	343.21^b^	343.97^b^	343.59^b^
P3	308.36^bc^	312.24^bc^	310.30^bc^	34.36^a^	35.49^abc^	34.93^ab^	341.58^b^	342.33^c^	341.96^c^
P4	306.52^ cd^	311.63^ cd^	309.08^ cd^	33.84^a^	34.78^abc^	34.31^ab^	339.34^c^	340.49^d^	339.92^d^
P5	304.78^de^	308.91^de^	306.84^de^	33.25^a^	34.02^bc^	33.64^ab^	336.77^d^	338.01^e^	337.39^e^
P6	302.10^e^	307.62^e^	304.86^e^	32.75^a^	33.44^c^	33.10^b^	337.21^d^	337.50^e^	337.36^e^
Significance	*	*	*	*	*	*	*	*	*
SE	0.37	0.46	0.30	0.26	0.16	0.15	0.59	0.33	0.39
Fertilizer modules									
FM1	300.69^b^	304.54^b^	302.62^b^	32.10^b^	33.00^b^	32.55^b^	332.87^b^	334.07^b^	333.47^b^
FM2	314.12^a^	319.56^a^	316.84^a^	36.19^a^	37.53^a^	36.86^a^	348.66^a^	348.91^a^	348.79^a^
Significance	*	*	*	*	*	*	*	*	*
SE	0.63	0.80	0.51	0.44	0.27	0.26	1.02	0.58	0.68
Interactions									
P1xFM1	305.48	309.21	307.35	33.66	35.09	34.38	338.36	339.76	339.06
P2xFM1	304.08	308.06	306.08	33.07	34.36	33.72	335.43	337.44	336.44
P3xFM1	302.52	305.24	303.88	32.27	33.05	32.66	335.05	336.19	335.62
P4xFM1	300.45	304.99	302.72	31.78	32.47	32.13	331.51	332.74	332.13
P5xFM1	297.59	300.40	299.00	31.13	31.69	31.41	328.66	329.76	329.21
P6xFM1	294.04	299.33	296.68	30.71	31.33	31.02	328.19	328.53	328.37
P1xFM2	318.23	324.50	321.37	37.87	39.78	38.83	354.60	353.52	354.06
P2xFM2	317.61	322.01	319.81	36.74	38.46	37.60	350.98	350.51	350.74
P3xFM2	314.19	319.24	316.71	36.44	37.93	37.19	348.12	348.47	348.30
P4xFM2	312.59	318.27	315.43	35.90	37.08	36.49	347.16	348.24	347.70
P5xFM2	311.96	317.41	314.69	35.38	36.36	35.87	344.87	346.25	345.56
P6xFM2	310.15	315.91	313.04	34.80	35.56	35.18	346.22	346.47	346.34
Significance	NS	NS	NS	NS	NS	NS	NS	NS	NS
SE	0.90	1.13	0.72	0.63	0.38	0.36	1.45	0.81	0.96

The values in each column that are preceded by the same letter are not significantly different from one another (DMRT, *p* ≤ 0.05)

Where *SE* is standard error, *NS* is Non Significant and * is significant at 5% significance.

Fertilization modules significantly affected viable microbial count of the soil. FM_1_ recorded the maximum viable bacterial (131.65 × 10^7^ cfu g^−1^ soil), viable actinomycetes (60.89 × 10^2^ cfu g^−1^ soil) and viable fungal (31.11 × 10^2^ cfu g^−1^ soil) count in soil, whereas, minimum (107.71 × 10^7^ cfu g^−1^ soil, 60.89 × 10^2^ cfu g^−1^ soil and 29.85 × 10^2^ cfu g^−1^ soil, respectively) was noted in FM_2_. P_1_ planting and FM_1_ resulted in a higher number of viable microbial count in the soil.

#### Soil biological activity

Soil microbial biomass varied significantly for planting dates and fertilization modules, however, interaction had non-significant effect. P_1_ exhibited the highest mean soil microbial biomass carbon content (48.79 µg g^−1^). Conversely, the lowest microbial biomass carbon content (47.03 µg g^−1^ soil) was registered in treatment P_6_ (47.03 µg g^−1^ soil). FM_1_ observed maximum soil microbial biomass (51.27 µg g^−1^ soil) in comparison to FM_2_ (44.86 µg g^−1^ soil) (Table 8).

**Table 8:** Effect of planting dates and fertilization on soil chemical properties

	pH			EC (ds m^−1^)			OC (%)		
Treatments	2021–22	2022–23	Pooled Mean	2021–22	2022–23	Pooled Mean	2021–22	2022–23	Pooled Mean
Planting Dates									
P1	6.87	6.89	6.88	0.41	0.40	0.40	1.11^a^	1.14^a^	1.13^a^
P2	6.87	6.88	6.88	0.39	0.38	0.39	1.09^ab^	1.12^ab^	1.11^ab^
P3	6.95	6.88	6.92	0.41	0.40	0.41	1.08^ab^	1.11^ab^	1.10^ab^
P4	6.88	6.88	6.89	0.38	0.38	0.38	1.09^ab^	1.10^ab^	1.09^ab^
P5	6.84	6.86	6.85	0.38	0.37	0.38	1.06^bc^	1.07^bc^	1.07^bc^
P6	6.92	6.87	6.89	0.37	0.37	0.37	1.04^c^	1.06^c^	1.05^c^
Significance	NS	NS	NS	NS	NS	NS	*	*	*
SE	0.02	0.02	0.01	0.01	0.01	0.01	0.01	0.01	0.01
Fertilizer modules									
FM1	6.88	6.88	6.88	0.39	0.38	0.39	1.11^a^	1.14^a^	1.13^a^
FM2	6.90	6.87	6.89	0.39	0.38	0.39	1.04^b^	1.07^b^	1.05^b^
Significance	NS	NS	NS	NS	NS	NS	*	*	*
SE	0.03	0.03	0.02	0.01	0.01	0.01	0.01	0.01	0.01
Interactions									
P1xFM1	6.87	6.87	6.87	0.40	0.39	0.40	1.15	1.17	1.16
P2xFM1	6.86	6.86	6.86	0.39	0.38	0.39	1.12	1.16	1.14
P3xFM1	6.93	6.90	6.92	0.40	0.40	0.40	1.10	1.15	1.13
P4xFM1	6.85	6.88	6.87	0.39	0.38	0.39	1.13	1.14	1.14
P5xFM1	6.84	6.87	6.86	0.37	0.39	0.38	1.10	1.11	1.11
P6xFM1	6.91	6.88	6.89	0.36	0.36	0.37	1.07	1.10	1.08
P1xFM2	6.87	6.90	6.89	0.41	0.40	0.41	1.08	1.11	1.09
P2xFM2	6.88	6.89	6.89	0.39	0.38	0.39	1.06	1.08	1.07
P3xFM2	6.97	6.86	6.92	0.42	0.39	0.41	1.06	1.08	1.07
P4xFM2	6.92	6.88	6.90	0.37	0.37	0.37	1.04	1.06	1.05
P5xFM2	6.85	6.85	6.85	0.39	0.36	0.38	1.02	1.03	1.02
P6xFM2	6.92	6.86	6.89	0.37	0.38	0.38	1.00	1.03	1.02
Significance	NS	NS	NS	NS	NS	NS	NS	NS	NS
SE	0.05	0.05	0.03	0.01	0.01	0.01	0.01	0.01	0.01

Where SE is standard error, NS is Non Significant and * is significant at 5% significance. The values in each column that are preceded by the same letter are not significantly different from one another (DMRT, *p* ≤ 0.05)

#### Soil enzymes

Data on soil dehydrogenase and phosphatase enzyme varied significantly for planting dates and fertilization modules, however, interaction had non-significant effect. Planting P_1_ demonstrated the highest mean activity levels of soil dehydrogenase enzyme (3.64 mgTPF h^−1^ g^−1^ soil), while the lowest value (2.99 mg TPF h^−1^ g^−1^ soil) was observed in P_6_. FM_1_ recorded the highest value of dehydrogenase enzyme in soil (3.77 mgTPF h^−1^ g^−1^ soil) in contrast to FM_2_ (2.92 mgTPF h^−1^ g^−1^ soil).

Similarly, phosphatase enzyme levels showed the highest values (23.79 mol PNP h^−1^ g^−1^ soil) in P_1_ and the lowest (17.33 mol PNP h^−1^ g^−1^ soil) in P_6_. FM_1_ recorded the highest value of phosphatase enzyme in soil (21.72 mol PNP h^−1^ g^−1^ soil) in comparison to FM_2_ (18.98 mol PNP h^−1^ g^−1^ soil) (Table 8). P_1_ planting and FM_1_ resulted in the highest enzyme population in soil.

### Pearson correlation analysis of *chrysanthemum* yield and quality parameters with soil properties

**Fig. 2:**
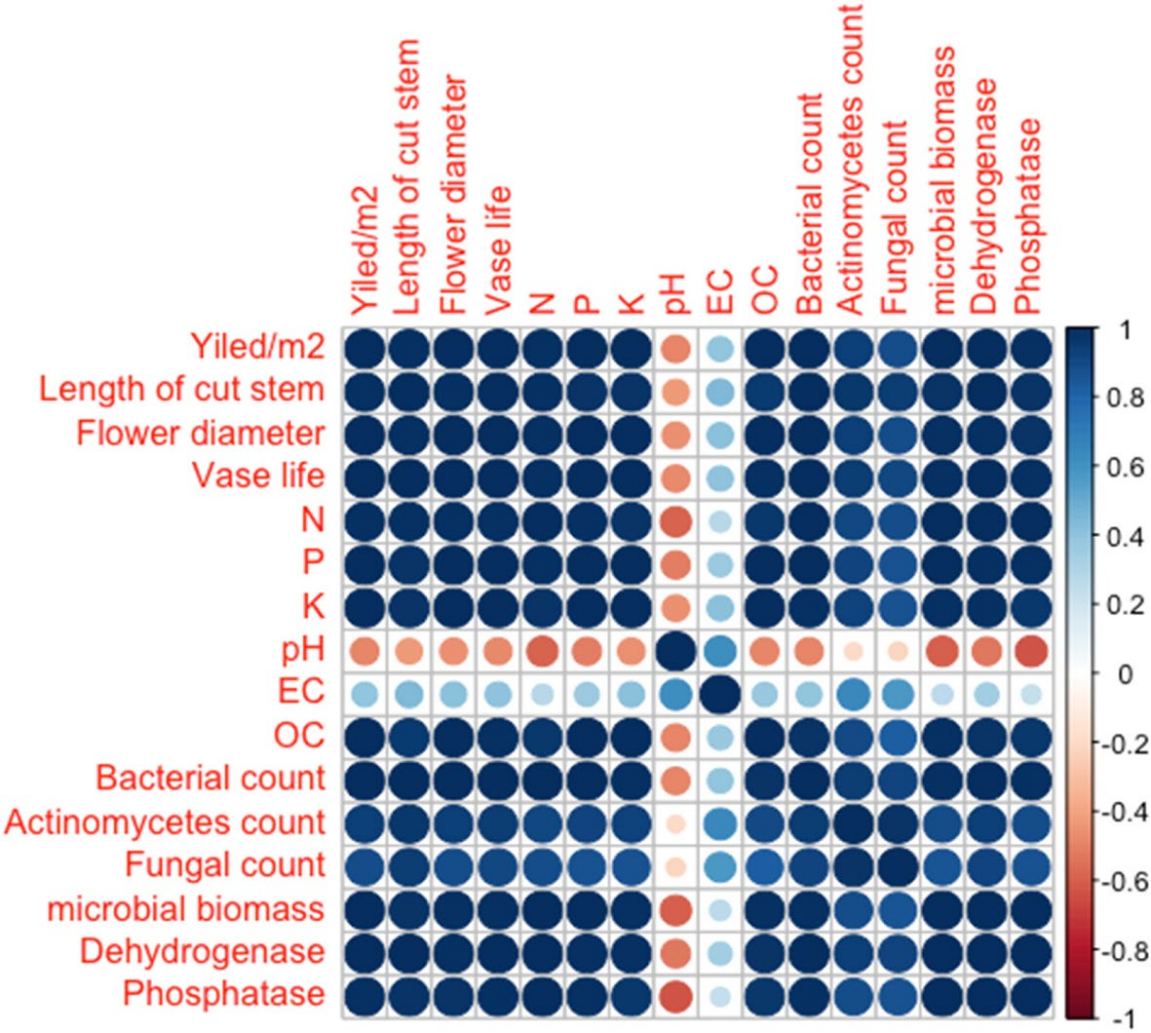
Correlation of cut stem yield and quality parameters with soil properties

Yield, length of cut stem, flower diameter and vase life has positive correlation with the macronutrient content of soil, organic carbon, microbial biomass, dehydrogenase and phosphatase enzymes, except for soil pH and soil EC. The available soil phosphorus exhibited the maximum correlation (r = 0.999) with the cut stem yield. The length of the cut stem has a maximum correlation (r = 0.993) with the dehydrogenase enzyme. Flower diameter has the maximum correlation (r = 0.999) with the soil available K and vase life has the highest correlation (r = 0.996) with the soil available P (Fig. 2).

#### Linear regression analysis for *chrysanthemum* flower diameter and vase life under different planting dates

**Fig. 3:**
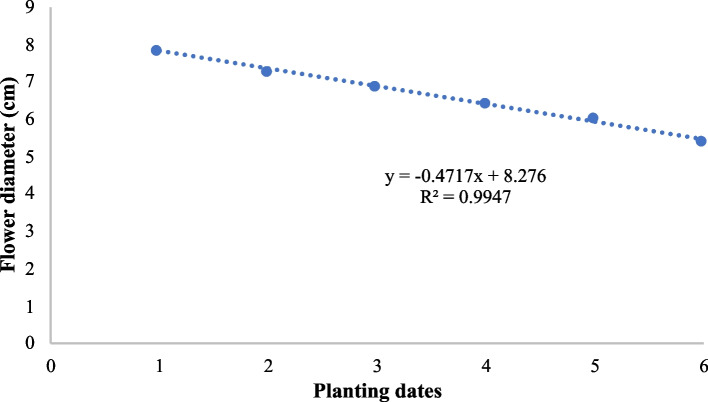
Linear regression analysis for flower diameter in chrysanthemum under different planting dates

In Fig. 3, the negative slope (-0.4717) indicates a negative correlation between planting dates and flower diameter. This means that later planting dates are associated with smaller flower diameters in chrysanthemums. The R^2^ value of 0.9947 suggesting that 99.47% of the variation in flower diameter can be explained by the planting dates. This indicates a very strong linear relationship between the two variables, this analysis implies that planting chrysanthemums earlier results in larger flowers, whereas delaying planting results in smaller flowers. This information can be crucial for planning planting schedules to achieve desired flower sizes.

**Fig. 4:**
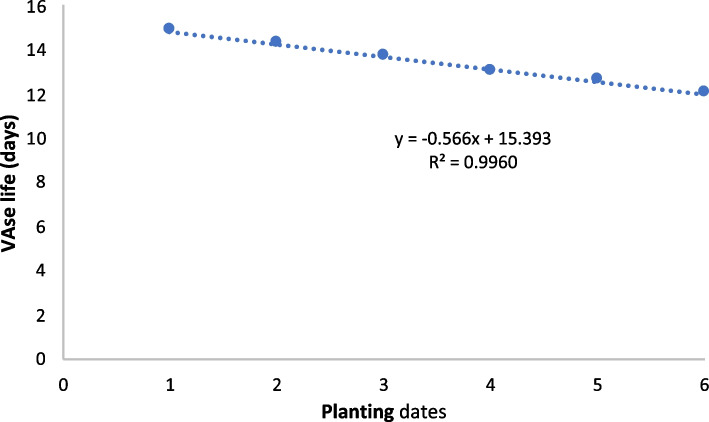
Linear regression analysis for vase life in chrysanthemum under different planting dates

In Fig. 4, the negative slope (-0.5660) indicates a negative correlation between planting dates and vase life. This means that later planting dates are associated with smaller vase life in chrysanthemums. The R^2^ value of 0.9960 suggesting that 99.60% of the variation in vase life can be explained by the planting dates. This indicates a very strong linear relationship between the two variables, this analysis implies that planting chrysanthemums earlier results in longer vase life, whereas delaying planting results in shorter vase life.

## Discussion

### Vegetative parameters

As the duration of daylight falls below a critical limit, the growth slows down. Hence, crops planted earlier received a more extended period of optimal long-day conditions conducive to better vegetative growth compared to those planted later, influencing the growth characteristics in response to planting dates (Table 4). In a similar study, improved vegetative characters viz., plant height and plant spread were highest in early plantings of *Chrysanthemum* and the minimum days required for bud formation and flowering were recorded with altered planting [26]. Likewise, results were reported by [27] in *Chrysanthemum* cv. ‘Aparajita’.

The difference due to fertilization may be attributed to the rapid availability of essential nutrients, particularly nitrogen, phosphorus and potassium in inorganic fertilizers, facilitating more immediate uptake and utilization by the plants [28]. In contrast, the organic fertilization method involving Jeevamrit, although offering nutrient richness but may have slower nutrient release rates and variability in nutrient composition in comparison to inorganic nutrient sources, potentially leading to slower nutrient uptake, consequently influencing plant growth to a slightly lesser extent [29]. All the vegetative parameters are greatly influenced by the application of N,P and K in *Chrysanthemum* [30] and marigold [31].

### Flowering, yield and quality attributes

Delayed planting necessitated fewer days for bud formation and flowering. This may be attributed to the fact that early planting led to an extended juvenile period, consequently requiring a longer duration for the initiation of flower bud formation and subsequent flowering in *Chrysanthemum* [24, 25] (Table 4). Planting *Chrysanthemum* earlier allows for an extended vegetative phase, fostering more branch development and, consequently, a greater number of flowering stems (Table 6). Conversely, delayed planting shortens the vegetative period due to decreasing daylight, leading to a quicker transition to flowering and potentially limiting branch growth, thereby reducing stem production [32]. However, in subsequent plantings (5 and 6), despite the late timing, the maximum stem count was achieved owing to higher plant density. The increased density in later plantings contributed to a more abundant yield of cut stems compared to earlier plantings, compensating for the shorter vegetative phase and ultimately resulting in higher stem production (Table 6). When planted earlier, chrysanthemum plants experience a longer duration of vegetative growth, allowing for greater energy accumulation and allocation towards floral development resulting in better flowering attributes [33]. This prolonged period also enables more robust flower bud differentiation and enlargement, resulting in larger flower sizes, greater stem length, improved vase life, duration of flowering and stem strength (Table 6). In contrast, later plantings, influenced by shorter day lengths, tend to expedite the transition to flowering, potentially limiting the time available for optimal flower and stem development, thus resulting in not-so-good flowers. Comparable findings have been documented by [34, 35] for *Chrysanthemum*

The application of NPK led to the accumulation of essential macro nutrients in the soil. Nitrogen, a component of chlorophyll, likely increased photosynthate synthesis, thereby enhancing plant vigour [36]. Phosphorus, another crucial nutrient, is integral to cellular proteins and nucleic acids, potentially promoting meristematic activity and resulting in increased stem length [37]. Additionally, potassium, a vital nutrient, activates enzymes crucial for protein and carbohydrate metabolism, fostering overall plant health and growth, enabling better resilience to adverse climatic conditions [38]. Thus, the combined application of NPK has proven better for high yielding attributes. The highest number of flowers per hectare was similarly observed in chrysanthemum plants when NPK was applied at a rate of 200:150:100 kg ha^−1^ [39]. Jeevamrit also have potentially contributes on enhancing overall plant health and vigor. It contains beneficial microorganisms and organic compounds that aid in improved nutrient uptake, physiological processes and defence mechanisms, ultimately supporting post-harvest quality and resilience, resulting in an extended vase life and improved yield characteristics [40].

### Macro-nutrient content and other chemical properties of soil

The current investigations found that the addition of nutrients from various sources and planting dates had a substantial impact on the chemical properties of the soil, with the exception of soil pH and EC. Inorganic fertilizers typically contain nitrogen in highly soluble forms, such as ammonium or nitrate, which are readily accessible for plant uptake, consequently increasing immediate soil nitrogen availability [41]. Moreover, the chemical composition of inorganic fertilizers facilitates a controlled and efficient release of nitrogen, ensuring a sustained supply over time [42]. Additionally, these fertilizers can include additives that enhance nitrogen retention and reduce losses due to leaching or volatilization, further increasing the amount of nitrogen available for plant utilization in the soil. Inorganic fertilizers usually contain phosphorus in forms that readily dissolve in soil moisture, making them more accessible to plant roots and thus increasing immediate soil phosphorus availability [43]. Additionally, the chemical structure of inorganic fertilizers allows for a controlled release of phosphorus, ensuring a sustained supply for plant uptake over time. Furthermore, inorganic fertilizers may contain additives or compounds that improve phosphorus solubility, reducing the fixation of phosphorus in the soil and enhancing its availability for plants [44]. Inorganic fertilizers typically contain potassium in forms that readily dissolve in soil water, increasing the immediate availability of potassium for plant uptake [45]. However, both modules showed an increase in available nitrogen, phosphorous and potassium indicating the fertilization had a positive effect on nutrient availability (Table 5).

The increased vegetative phase in earlier plantings enables greater root development, facilitating better nutrient uptake and utilization from the soil, thus resulting in higher available NPK levels compared to later plantings [46].

Overall, the findings indicate that both inorganic and organic fertilization had positive effects on soil parameters and nutrient availability. The inorganic fertilization module showed slightly higher values for pH, electrical conductivity, available nitrogen, phosphorus and potassium compared to the organic fertilization module. The organic fertilization module, on the other hand, resulted in a slightly higher organic carbon content. These results suggest that both types of fertilizers can contribute to soil fertility, but the specific choice of fertilizer depends on various factors such as crop requirements, environmental considerations and sustainable farming practices (Table 6). These results are in conformity with the results of [47].

Bio-enhancer preparations incorporate effective microorganisms serving as bio-inoculums, facilitating mineralization processes and restoring soil fertility. Biostimulants contain significant reserves of carbon and mineral nutrients, which are released as microorganisms decompose them [48]. Soybean, field bean, corn and paddy microbial populations were significantly impacted by fermented organic liquid inputs [49]. Applying varied concentrations of panchagavya to the seeds of grains and legumes, such as soybean, pea, black gram, green gram, moth bean, dry bean and lentil, increased the microbiological activity of the soil [50]. The results also showed that soil application of biostimulants activated the microorganisms in the rhizosphere of chrysanthemum plants. Total microbial counts were increased in the rhizosphere of plants treated with Jeevamrit compared to the inorganic fertilization module (Table 9). Increased microbial counts may be due to nutrient availability in the rhizosphere of Jeevamrit treated plants, which provide the needed energy for soil microorganisms to decompose organic matter [51]. Since beejamrit, jeevamrit and panchagavya are derived from cow products, they have large quantities of beneficial microorganisms like fungus, actinomycetes, methylotrophs, Azotobacter, Pseudomonas, lactic acid bacteria, Phosphobacteria and Azospirillum [52]. Simple sugars and other readily biodegradable substances are indicated by the presence of enzyme activity in diverse organic inputs. Increases in soil enzyme activity (Table 10), which is influenced by edaphic characteristics, crop type, cultivation methods, climate, and conditioning, are markers of soil fertility. Because dehydrogenases are found in both soil and living cells, where they accelerate oxidoreductive reactions, they are associated with soil microbiological activity. The enzymatic activity, interconnected with humic substances, soil colloids, as well as plants, living cells, deceased cells and microorganisms, is associated with soil phosphatase activity [53]. The addition of panchagavya, beejamrit, jeevamrit, and FYM to chilli produced a notable increase in the soil's dehydrogenase activity. The findings also align with the results of [54] who noted that utilizing arbuscular mycorrhizal fungi and plant growth-promoting rhizobacteria can boost soil enzymatic activity and improve soil microbial population in the rhizosphere of guar plants, ultimately increasing yield. Similar results of improved soil health with the application of bio and organic fertilizers have been reported by [55] in hybrid maize (*Zeamays* L.).The collective impact of amendments influences the soil's ability to provide nutrients to plants by affecting the turnover of organic matter in the soil. This, in turn, has repercussions on the soil microbial biomass—an integral component of soil organic matter serving as a labile reservoir for plant-available nitrogen (N), phosphorus (P), and sulfur (S) [56]. The utilization of organic manures (FYM), neem cake, rock phosphate, and biofertilizers (Azotobacter) in a paddy field resulted in a significant increase in soil microbial biomass-C [57, 58].

**Table 9.** Effect of planting dates and fertilization on soil microbiological properties

	Bacterial Count (× 10^7^ cfu g^−1^ soil)			Actinomycetes Count (× 10^2^ cfu g^−1^ soil)			Fungal Count (× 10^2^ cfu g^−1^ soil)		
Treatments	2021–22	2022–23	Pooled Mean	2021–22	2022–23	Pooled Mean	2021–22	2022–23	Pooled Mean
Planting Dates									
P1	121.23^a^	128.51^a^	124.87^a^	60.23	61.19	60.72	30.83	31.08	30.95
P2	119.64^ab^	126.19^ab^	122.92^ab^	59.93	60.82	60.37	30.81	31.05	30.94
P3	117.04^abc^	124.11^abc^	120.57^abc^	59.70	60.61	60.16	30.67	30.89	30.79
P4	115.65^abc^	121.04^abc^	118.35^abc^	58.73	59.62	59.18	30.31	30.54	30.43
P5	114.03^bc^	118.95^bc^	116.49^bc^	58.22	59.19	58.70	30.20	30.47	30.34
P6	112.60^c^	117.20^c^	114.90^c^	57.96	58.89	58.43	29.31	29.54	29.43
Significance	*	*	*	NS	NS	NS	NS	NS	NS
SE	0.80	0.56	0.23	0.93	0.58	0.23	0.82	0.57	0.23
Fertilizer modules									
FM1	128.30^a^	135.00^a^	131.65^a^	60.40^a^	61.37^a^	60.89^a^	30.99^a^	31.23^a^	31.11^a^
FM2	105.09^b^	110.33^b^	107.71^b^	57.85^b^	58.74^b^	58.30^b^	29.73^b^	29.96^b^	29.85^b^
Significance	*	*	*	*	*	*	*	*	*
SE	1.38	0.97	0.40	1.60	1.00	0.39	1.43	0.98	0.40
Interactions									
P1xFM1	133.74	140.91	137.32	61.41	62.48	61.95	30.20	30.45	30.33
P2xFM1	131.45	138.95	135.20	61.01	61.98	61.50	30.18	30.40	30.29
P3xFM1	128.64	135.38	132.01	60.81	61.70	61.26	30.06	30.26	30.16
P4xFM1	126.77	134.16	130.46	59.92	60.84	60.39	29.69	29.93	29.82
P5xFM1	125.63	132.47	129.05	59.83	60.86	60.35	29.55	29.83	29.69
P6xFM1	123.57	128.15	125.86	59.44	60.37	59.91	28.68	28.89	28.79
P1xFM2	108.71	116.11	112.41	59.05	59.91	59.49	31.45	31.71	31.58
P2xFM2	107.83	113.43	110.63	58.84	59.65	59.25	31.44	31.70	31.58
P3xFM2	105.43	112.83	109.13	58.60	59.53	59.07	31.28	31.52	31.41
P4xFM2	104.53	107.92	106.23	57.53	58.41	57.97	30.93	31.15	31.04
P5xFM2	102.43	105.43	103.93	56.60	57.51	57.06	30.85	31.11	30.98
P6xFM2	101.62	106.25	103.94	56.48	57.41	56.94	29.95	30.18	30.07
Significance	NS	NS	NS	NS	NS	NS	NS	NS	NS
SE	1.95	1.37	0.57	2.27	1.42	0.55	2.02	1.39	0.56

The values in each column that are preceded by the same letter are not significantly different from one another (DMRT, *p* ≤ 0.05)

Where *SE* is standard error, *NS* is Non Significant and * is significant at 5% significance

**Table 10.** Effect of planting dates and fertilization on soil microbiological properties

	Microbial Biomass (µg g^−1^)			Dehydrogenase Enzyme (mg TPF h^−1^ g^−1^ soil)			Phosphatase Enzyme (mmole PNP h^−1^ g^−1^ soil)		
Treatments	2021–22	2022–23	Pooled Mean	2021–22	2022–23	Pooled Mean	2021–22	2022–23	Pooled Mean
Planting Dates									
P1	47.73^a^	49.84^a^	48.79^a^	3.54^a^	3.73^a^	3.64^a^	23.43^a^	24.14^a^	23.79^a^
P2	47.50^ab^	49.56^ab^	48.53^ab^	3.45^a^	3.65^a^	3.55^a^	22.21^a^	23.06^a^	22.64^a^
P3	47.19^ab^	49.20^ab^	48.20^ab^	3.27^ab^	3.54^ab^	3.41^ab^	19.96^ab^	20.99^ab^	20.48^ab^
P4	47.02^ab^	49.09^ab^	48.06^ab^	3.17^ab^	3.44^ab^	3.31^ab^	19.17^ab^	20.33^ab^	19.75^ab^
P5	46.80^ab^	48.76^ab^	47.79^ab^	3.04^ab^	3.29^ab^	3.17^ab^	17.76^b^	18.51^b^	18.14^b^
P6	45.99^b^	48.07^b^	47.03^b^	2.87^b^	3.10^b^	2.99^b^	16.82^b^	17.83^b^	17.33^b^
Significance	*	*	*	*	*	*	*	*	*
SE	0.21	0.21	0.21	0.07	0.06	0.06	0.57	0.59	0.58
Fertilizer modules									
FM1	50.26^a^	52.28^a^	51.27^a^	3.66^a^	3.87^a^	3.77^a^	21.38^a^	22.06^a^	21.72^a^
FM2	43.82^b^	45.89^b^	44.86^b^	2.79^b^	3.04^b^	2.92^b^	18.41^b^	19.56^b^	18.98^b^
Significance	*	*	*	*	*	*	*	*	*
SE	0.36	0.37	0.36	0.11	0.11	0.11	0.98	1.03	1.00
Interactions									
P1xFM1	50.73	52.77	51.75	3.92	4.13	4.03	25.39	25.82	25.60
P2xFM1	50.69	52.69	51.69	3.85	4.02	3.94	24.46	25.03	24.75
P3xFM1	50.40	52.45	51.43	3.72	3.94	3.83	20.82	21.65	21.24
P4xFM1	50.31	52.37	51.34	3.51	3.73	3.62	19.81	20.62	20.22
P5xFM1	50.12	51.98	51.05	3.47	3.69	3.59	19.59	20.11	19.85
P6xFM1	49.29	51.40	50.35	3.46	3.70	3.59	18.19	19.15	18.67
P1xFM2	44.72	46.91	45.82	3.17	3.34	3.25	21.47	22.47	21.97
P2xFM2	44.32	46.42	45.37	3.04	3.27	3.16	19.97	21.10	20.53
P3xFM2	43.97	45.95	44.96	2.82	3.14	2.98	19.10	20.32	19.71
P4xFM2	43.73	45.81	44.77	2.83	3.14	2.99	18.52	20.04	19.28
P5xFM2	43.49	45.54	44.52	2.60	2.88	2.74	15.94	16.90	16.42
P6xFM2	42.70	44.73	43.72	2.28	2.49	2.39	15.45	16.51	15.98
Significance	NS	NS	NS	NS	NS	NS	NS	NS	NS
SE	0.51	0.52	0.51	0.16	0.15	0.16	1.39	1.45	1.42

The values in each column that are preceded by the same letter are not significantly different from one another (DMRT, *p*≤0.05)

Where *SE* is standard error, *NS* is Non Significant and * is significant at 5% significance

### Correlation analysis

The positive correlation observed between the cut stem yield, length of cut stem, flower diameter, and vase life of chrysanthemum with various soil macronutrients, organic carbon, microbial biomass, dehydrogenase, and phosphatase enzymes can be attributed to the intricate interplay between soil health and plant growth (Fig. 2). Adequate levels of macronutrients, including nitrogen, phosphorus, and potassium, provide essential elements for plant development, influencing stem growth, flower size, and overall yield. Organic carbon contributes to soil structure, water retention, and nutrient availability, promoting robust root systems and, consequently, enhancing floral attributes. Microbial biomass, dehydrogenase, and phosphatase enzymes are indicators of soil microbial activity, playing crucial roles in organic matter decomposition, nutrient cycling and nutrient release for plant uptake. The positive correlation underscores the significance of a well-balanced and biologically active soil environment in fostering optimal conditions for chrysanthemum growth, leading to improved stem yield, length, flower diameter, and vase life. Moreover, the organic amendments facilitate the gradual release of nutrients, which plants utilize gradually, contributing to increased yields and improved nutrient levels in the soil [10, 59]. Varying planting dates influence soil temperature and moisture, affecting microbial activity and nutrient release.

## Conclusion

This field study on planting dates and fertilizer modules provides crucial insights for enhancing *Chrysanthemum* cultivation. Early planting (P_1_) accelerates growth stages and improves flower quality, yield and soil health. Additionally, applying NPK at 30 g m^−2^ enhances bud formation, plant vitality and soil fertility. These findings are significant for *Chrysanthemum* growers, guiding them towards informed decisions and sustainable practices. Moving forward, integrating precision agriculture, adapting to climate change, exploring organic fertilizers, monitoring soil health, and facilitating knowledge transfer will further promote environmentally friendly and resilient *Chrysanthemum* cultivation.

## Data Availability

Data is provided within the manuscript. Any other information on datasets used in this study are available from the corresponding author upon request.
